# Spontaneous and Electrically Induced Anisotropy of Composite Agarose Gels

**DOI:** 10.3390/gels8110753

**Published:** 2022-11-21

**Authors:** Alexandar M. Zhivkov, Svetlana H. Hristova

**Affiliations:** 1 Institute of Physical Chemistry, Bulgarian Academy of Sciences (BAS), Acad. G. Bonchev Str., bl. 11, 1113 Sofia, Bulgaria; 2 Department of Medical Physics and Biophysics, Medical Faculty, Medical University—Sofia, Zdrave Str. 2, 1431 Sofia, Bulgaria

**Keywords:** agarose gels, bacteriorhodopsin, purple membranes, optical anisotropy, electric birefringence

## Abstract

Agarose gels containing and not bacteriorhodopsin purple membranes (incorporated before gelling) manifest spontaneous optical anisotropy. The dependencies of the anisotropy on the agarose concentration and time have been studied. The rise in the anisotropy is explained by the predominant orientation of the agarose fibers during the gelling and subsequent deformation of the gel net. In the electric field, additional optical anisotropy rises, which is caused by the orientation of the membranes. A procedure has been developed to separate electrically induced and spontaneous anisotropy in composite gels. The isoelectric points and surface electric potential of bacteriorhodopsin trimer and purple membranes are calculated by the method of protein electrostatics to explain their electric asymmetry, which leads to perpendicular orientation in the direct electric field and longitudinal in the kilohertz sinusoidal field. The results allow for an increase in the separation capability of composite gels of electrophoresis for macromolecules with different sizes by applying an appropriate electric field to modulate the effective pore size.

## 1. Introduction

### 1.1. Agarose Gels in Direct Electric Field

Agarose is widely used as a carrier in the gel-electrophoresis of DNA [1], as well as for the separation of high molecular weight dsDNA using the method of pulsed field electrophoresis [2]. The agarose gel structure is very heterogeneous and contains large interstitial spaces bounded by fibrous areas of varying densities [3,4]. In order to study the mechanism of gel-electrophoresis, investigations were carried out measuring the electrophoretic mobility of particles of varying origins, form and size: DNA [5,6,7], viruses [8,9], latex particles [10,11] in d.c. [8] and pulse [5,12,13] electric fields. The conclusions were drawn based on the assumption that there is no aggregation and the fractions of a given particle size in the gel are the same as those in the suspension before adding the solution of melted agarose. Even when the particles are monodispersed, the main problem is that the pores are very different in size because of the inhomogeneous structure of the gels. Therefore, the aggregates and larger particles are caught in smaller pores and cannot be moved by the applied d.c. electric field; only the smallest particles migrate. Therefore, the methods based on the translational mobility of particles in the direct or pulse electric field provide distorted information of the pore size.

### 1.2. Agarose Gels in Alternative Electric Field

To avoid above imperfections, we applied a different approach based on the rotational mobility of non-spherical particles in the sinusoidal electric field that orient them without translational movement. Therefore, we prepared composite agarose gels by adding a suspension of suitable particles to melt agarose solutions before the casting; as they are incorporated in the gel beforehand, the particles are evenly distributed in volume. 

To monitor the orientational behavior of the particles, we used electrooptical methods [14], which are based on the change in the optical properties of a disperse system of anisodiametric particles due to their orientation when applying an electric field with suitable strength and frequency. In the present investigation, we employed the techniques of electric birefringence [15], which is based on induced optical anisotropy (different refractive indexes along and across the electric field) of the gel, which arise due to particle orientation. This leads to elliptical polarization of linearly polarized light beams that transmit across the specimen. The main advantage of this technique is that it allows for the investigation of both the composite and clear agarose gels (the method of electric dichroism is only applicable to composite gels with light absorbing particles).

Extensive investigations of agarose gels containing dsDNA fragments using the method of electric birefringence have been carried out by Stellwagens [5,6,16,17,18,19,20,21,22]. In clear agarose gels, they detected both positive and negative electrooptical effects (EOE) with second kinetics of relaxation after the application of rectangular electric impulses: Short high voltage (1–10 kV/cm, 0.02–1 ms) or long low voltage (5–15 V/cm, 0.5–15 s). The effects were explained by the orientation of microscopic domains and fiber bundles. Other than the electrically-induced birefringence, they found a spontaneous one. The last is an indication of intrinsic optical anisotropy. The authors suggest that the agarose gel matrix has a hierarchical structure of loosely connected substructures of microdomains and fiber bundles, held together by metastable hydrogen bonds. The sign and magnitude of both induced and intrinsic birefringence depend on the location in the gel; this means that the agarose gels are strongly heterogeneous. Such heterogeneity has also been found in other polysaccharide gels, namely highly charged agarose and β-carrageenan (stereoisomer of agarose), in contrast to the cross-linked chemical gels as polyacrylamide, which have a homogeneous internal structure.

Investigating agarose gels containing colloid particles, we encountered a major difficulty: The composite gels have spontaneous optical anisotropy, which does not allow for the application of the classical theory of electric birefringence. As the interpretation of the results depends substantially on the initial anisotropy, we studied the birefringence of specimens with and without incorporated particles and before and after electric field application. In the present article, we proposed a procedure to distinguish between the spontaneous and electrically induced optical anisotropy.

### 1.3. Purple Membranes

To prepare composite agarose gels, we used a monodispersed water suspension of purple membranes (PM), which are plate-like particles with a diameter of 0.3–1.2 μm and a thickness of 5 nm. The PM consist of membrane protein bacteriorhodopsin (bR) and lipids, which take 3/4 and 1/4 of the mass, respectively; 9/10 of the lipids are negatively charged. bR is a chromoproteid. One of its aminoacid residues is covalently bound with retinal, which provides a violet color of PM [23]. The absorption spectrum has a broad band with a maximum at 570 nm; the absorption in the range 350–470 nm is five times lower, and it is zero above 650 nm [24]. The refractive index of PM is 1.53 at 650 nm [25]. The polypeptide chain of bR forms seven α-helix segments that are orientated perpendicularly to the membrane surface. The bR macromolecules are grouped into trimers, which have cylindrical form and are densely packed by forming a two-dimensional (2D) hexagonal lattice with a 6.2 nm interpoint distance; the space between the trimers is filled by lipid molecules. This structural peculiarity provides unique properties: In difference from the other biological and artificial lipid membranes, PM are not closed in vesicles and by that, their structurally-determined electric asymmetry manifests itself as a permanent dipole moment that orients PM perpendicularly to the lines of force in the direct electric field. In a sinusoidal electric field, the orientation depends on the competition of the permanent and induced dipole moments across the field at frequencies under 100 Hz or along at kilohertz frequencies [26]. In the electric fields with a strength up to 2 kV/cm, PM behave as inflexible particles [27].

### 1.4. Electric Birefringence

In this investigation, the electric birefringence method [15] was employed to measure the spontaneous Δ*n*_0_ and electrically-induced Δ*n*_E_ optical anisotropy of agarose gels. The techniques are based on a rising of the electrooptical effect (EOE) Δ*I* = *I*_E_ − *I*_0_: an alteration in the intensity *I* of the light transmitting through suspension of non-spherical colloid particles in electrooptical cell at application of electric field with a strength *E* by two metal electrodes; EOE is caused by the orientation of the particles [14]. When the entering light beam is linearly polarized and the polarization plane is not parallel or perpendicular to the field direction (lines of force), it becomes elliptically polarized after passing through the suspension due to the different phase velocities of the two components of the refractive index *n*: parallel *n*^//^ and perpendicular *n*^⊥^ to the field direction; the difference determines the optical anisotropy Δ*n*_E_ = *n*^//^ − *n*^⊥^ at the applied electric field. The phase retardation δ of the two components (*n*^//^ > *n*^⊥^ at particle orientation along the electric field, then the parallel light component is slower) is determined by Δ*n*_E_, the wave length λ_0_ in vacuum (λ = λ_0_/*n* in the medium) and the optical path *l* in the electrooptical cell:

δ_E_ = 2π (*l/*λ_0_) Δ*n*_E_ (1)

The apparatus for electric birefringence includes a polarizer (P), analyzer (A) and electrooptical cell between them (Section 4.2). When the polarization plane of the entering beam is declined at 45° to the electric field, the intensity *I* of light passing through the analyzer depends on the intensity *I*_0_ of the incident light and the decrossing angle φ (φ = 0 at fully crossed polarizers, A⊥P) [15]:

*I =* (*I*_0_/2) [1 − cos(2φ) cos δ_E_] (2)

This equation can be rewritten with the absence of optical anisotropy (*E* = 0, δ_E_ = 0):

*I*_φ_ = *I*_0_ sin^2^φ (3)

and in the presence of electrically-induced anisotropy (*E* > 0, δ_E_ ≠ 0) at φ = 0 (A⊥P):

*I*_E_ = *I*_0_ sin^2^ (δ_E_/2) (4)

The combination of Equations (3) and (4) provides δ_E_ = 2φ at *I*_E_ = *I*φ; this equality allows for the determination of the electrical phase retardation δ_E_ if the intensity *I*_E_ of the light transmitting through the electrooptical cell at fully crossed polarizers (φ = 0) is caused only by electrically-induced optical anisotropy (Δ*n*_E_ ≠ 0) (in the absence of spontaneous anisotropy, Δ*n*_0_ = 0). That is, at the applied electric field with a strength *E,* the light intensity *I*_E_ at the crossed polarizers (A⊥P) is equal to *I*φ at some decrossing angles φ ≠ 0 when *E* = 0 (absence of particle orientation, δ_E_ = 0). The EOE (Δ*I* = *I*_E_ at φ = 0 and Δ*n*_0_ = 0) can be determined by equating *I*_E_ to *I*φ with the aid of the calibration graph *I*φ *= f*(sin^2^φ). Then, δ_E_ can be calculated using Equation (4), and finally, Equation (1) provides the electrically-induced optical anisotropy Δ*n_E_*:

Δ*n*_E_ = (λ_0_/2π*l*) δ_E_ (5)

The equality of *I*_E_ and *I*φ on the ordinate of the graph *I*φ *= f*(sin^2^φ) allows for measuring their values in relative units (millivolts in our case) instead of the absolute units of the light intensity [W/m^2^]. Then, the measured values *I*_E_ [mV] = *KI*_0_ sin^2^ (δ_E_/2) and *I*φ [mV] = *KI*_0_ sin^2^φ are proportional to the apparatus constant *K* (coefficient of multiplication), which is determined by the spectral sensitivity of the photocathode and power voltage of the photomultiplier used, where the photoanode current *I*_anode_ is proportional to *I*_E_ and to the voltage *I*_anode_ *R* [mV] measured on the anode resistor *R*.

## 2. Results and Discussion

The results and the main interpretations are described in the Section 2.1, Section 2.2, Section 2.3, Section 2.4, Section 2.5 and Section 2.6. The advanced interpretations are discussed in Section 2.7, Section 2.8, Section 2.9, Section 2.10, Section 2.11, Section 2.12, Section 2.13, Section 2.14, Section 2.15 and Section 2.16.

### 2.1. Purple Membranes

**Figure 1:**
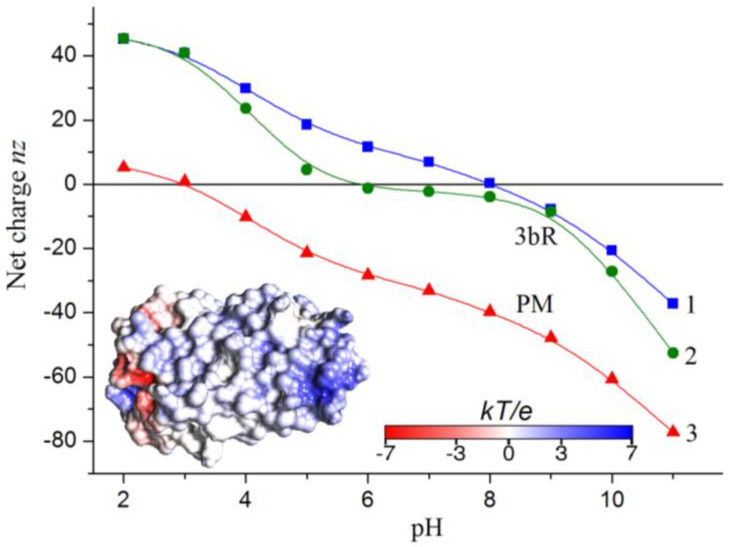
pH-dependences of the net charge *nz* of the polypeptide chain of bacteriorhodopsin trimer (3bR) in folded (curve 1) and unfolded (curve 2) conformation, and *nz* of purple membrane (PM, curve 3). *Insert*: Molecular model of single bR macromolecule horizontally oriented with the cytoplasmic side on the left. The colors correspond to the sign (red—negative and blue—positive) and the value of the electrostatic potential on the intramembrane surface of the bR monomer in the range of −7 *kT*/*e* to +7 *kT*/*e*, where *e*—the elementary charge, *k*—Boltzmann constant, and *T*—absolute temperature; *kT*/*e* = 25.26 mV at 20 °C.

Figure 1 shows the *nz*(pH) dependences of the net coulomb charge *nz* of the bacteriorhodopsin trimer (3bR) in aqueous medium. The *nz* is computed by the method of protein electrostatics in two forms: Denaturated (unfolded, a random coil chain) and native (folded), assuming that 3bR is a protein globule with three subunits, each with a three-dimensional (3D) structure of bR monomer with atomic coordinates corresponding to those of the native bR macromolecule (Protein data bank ID: 1BRR). The difference is that, in the folded state, part of the chargeable aminoacid residues are not ionized because of their inaccessibility to H_2_O molecules of the aqueous medium and the low dielectric permittivity of the hydrophobic intramembrane parts of the seven α-helix segments of the bR macromolecule. This difference in conformation leads to a shift in the isoelectric point pI by 2 pH units (from pI 5.9 to pI 8.0) at the folding of the 3bR polypeptide chain. The calculated value of the isoelectric point for the unfolded chain is close to the experimentally measured pI 5.2–5.6 at full denatured bR [28]. The *nz* (pH) dependence of PM is obtained by assuming that 30 lipid molecules are associated to one bR trimer, and they bear 40 negative charges (10 twinned molecules as cardiolipin have two charges). The negative charges of the lipid heads shift the isoelectric point of PM with 5 pH-units in comparison to the pI of 3bR: From pI 8.0 to pI 3.0. At neutral pH 6–7, the net charge *nz* of PM is negative due to the contribution the lipid molecules.

The surface electrostatic potential of the bR monomer and trimer (3bR) is computed by the techniques of protein electrostatics (Section 2.14) and visualized according to its value and sign: Negative in red, positive in blue. The potential on the intramembrane (hydrophobic) surface of the monomer (Figure 1, Insert) is close to zero because the charged aminoacid residues are located on the two hydrophilic sides of the protein globule. At pH 6.0, the positive potential slightly predominates; this result is in accordance with the *nz*(pH) dependence (curve 1 in Figure 1), which discloses that the net charge of the bR trimer is positive at pH 6–7. However, the potential is asymmetrically distributed. It is positive on the extracellular side of the bR monomer and negative on the intracellular side.

**Figure 2:**
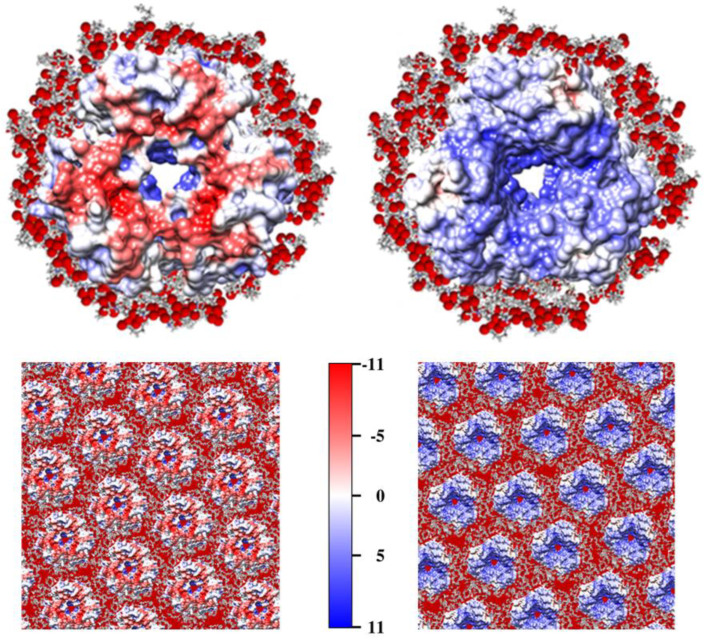
Molecular models of bacteriorhodopsin trimer (3bR) with adjoining 30 lipid molecules (the upper two pictures) and a part of the purple membrane (the lower two pictures). The left two pictures show the intracellular (cytoplasmic) surface of bR and PM and the right two pictures show the extracellular surface. The colors correspond to the sign and value of the electrostatic potential at pH 6.0: Negative (red) and positive (blue) in the range of −11 *kT*/*e* to +11 *kT*/*e*. The red points around the bR trimer represent the oxygen atoms in the heads of the lipid molecules.

Figure 2 (the two upper pictures) shows the two extramembrane (hydrophilic) surfaces of 3bR-lipid complex: bR trimer with 30 adjacent lipid molecules whose positions are obtained by the method of molecular dynamics. The protein/lipid ratio and kind of the lipid molecule in the model corresponds to the experimentally found ones [29]: one phosphatidylglycerophosphate methyl ester, three glycolipid sulfate, one phosphatidylglycerol, one archaeal glycocardiolipin, two squalene plus minor amounts of phosphatidylglycerosulfate (PGS) and two archaeal cardiolipin per one bR monomer; this lipid content means that the 30 lipid molecules carry about 40 negative charges per one bR trimer. The oxygen atoms in the heads of the lipid molecules are colored in red, considering their coulomb or partial negative charges.

Figure 2 (bottom two pictures) shows the two (hydrophilic) surfaces of PM, the model is constructed by the arrangement of bR trimers (conjunct with 30 lipid molecules per one 3bR) in a two-dimensional (2D) hexagonal lattice. The comparison of the two pictures shows that the electrostatic potential is asymmetrically distributed on the two surfaces of the PM due to the 3D structural asymmetry (disposition of the charged aminoacid residues) of bR trimers whose positive charges are located predominately on their extracellular surface and the negative is on the intracellular (cytoplasmic) surface. In the direct electric field, this asymmetry manifests itself as a permanent dipole moment that orients PM with the more negatively charged intracellular surface to the anode, and with the extracellular surface to the cathode (Section 2.16).

### 2.2. Electric Birefringence in an Anisotropic Medium

The form of the electrooptical effect of PM incorporated in an agarose gel is deformed, as compared to that in the water suspension; therefore, it is not possible to determine Δ*n* using Equations (4) and (5) with a field strength dependence *I*_E_ = *f*(*E*^2^), as in the case of free particles in liquid medium where its orientation is unperturbed. As we have established, the anomalous electrooptical behavior of incorporated PM is due to the intrinsic (spontaneous) optic anisotropy Δ*n*_0_ of the gel. In order to separate the spontaneous from the electrically induced anisotropy Δ*n*_E_ (caused by PM orientation), we have developed the following approach, which allows us to distinguish the two component of the anisotropy using the classical experimental technique of electric birefringence. The approach is based on the presumption of the additivity of the spontaneous and electrically induced anisotropies.

When a spontaneous optical anisotropy Δ*n*_0_, resulting in phase lag δ_0_ in the gel that already exists, at applying of the electric field, an additional phase delay δ_E_ arises. The total phase retardation δ = δ_0_ + δ_E_ can be determined by measuring the light intensity *I*(0°) transmitting through the analyzer at φ = 0 (A⊥P) in the absence of field (*E* = 0) and its change Δ*I*_E_ when electric field *E* is applied:

*I*_E_ = *I*(0°) + Δ*I*_E_ = *KI*_0_ sin^2^ (δ/2) (6)

where *K* and *I*_0_ [mV] are the apparatus constant and the intensity of the incident light beam (entering in the electrooptical cell), respectively.

Constant *KI*_0_ [mV] and initial phase difference δ_0_ may be determined if we rewrite Equation (2) (δ_E_ = 0, δ = δ_0_) in the form:

*I*_φ_ = (*KI*_0_/2) (1 − cos δ_0_ + 2 cos δ_0_ sin^2^ φ) (7)

The slope of the graph *I*_φ_ = *f*(sin^2^φ) is equal to *KI*_0_cosδ_0_. From this slope, the initial ordinate *I*(0°) (at φ = 0), *KI*_0_ and δ_0_ can be determined:

*KI*_0_ = 2 *I*(0°) + *KI*_0_ cos δ_0_ (8)

cos δ_0_ = 1 − 2 *I*(0°)/*KI*_0_ (9)

The spontaneous anisotropy Δ*n*_0_ and electrically induced anisotropy Δ*n*_E_ of the refractive index *n* at λ_0_ are defined from Equation (1):

Δ*n*_0_
*=* (λ_0_/2π *l*) δ_0_ (10)

Δ*n*_E_ = (λ_0_/2π *l*) (δ − δ_0_) (11)

where:

δ = 2 arcsin{[*I*(0°) + Δ*I*]/*KI*_0_}^1/2^ (12)

Thus, the procedure for the determination of the spontaneous Δ*n*_0_ and induced Δ*n*_E_ components of the complex optical anisotropy Δ*n* is as follows:

1. Measure the intensity *KI*_0_ [mV] of light transmitted through the analyzer at the decrossing angle φ (in absence of electric field) and drawing the dependence *KI*_0_ = *f* (sin^2^ φ).

2. Determine the phase retardation δ_0_ according to Equation (9) from the initial ordinate *I*(0°) and the slope *KI*_0_ cosδ_0_ of the line *KI*_0_ = *f*(sin^2^φ) and the spontaneous optical anisotropy Δ*n*_0_ according to Equation (10).

3. Measure the electrooptical effect Δ*I*_E_ at φ = 0.

4. Determine the total phase retardation δ according to Equation (12) and the electrically induced optical anisotropy Δ*n*_E_ according to Equation (11).

### 2.3. Linear Birefringence of Composite Gels

**Figure 3:**
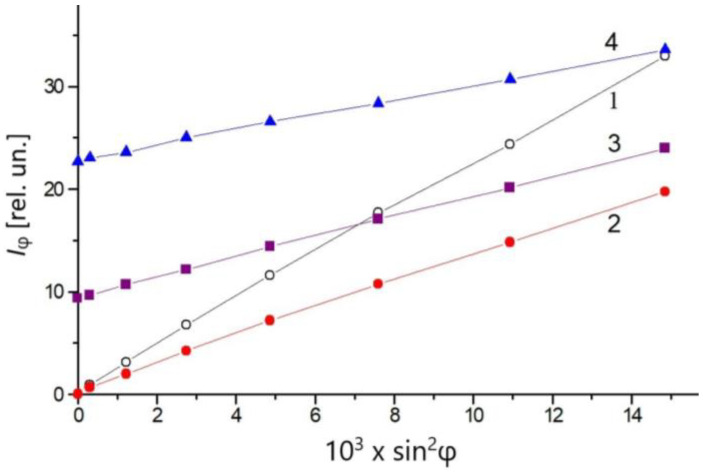
Dependence of the intensity *I*_φ_ of the transmitted light with a wavelength of 650 nm on the angle of decrossing φ (degrees) in the aqueous suspension of PM and in composite agarose gels with incorporated PM in equal concentrations: 1—PM in bidistilled water; 2—PM in fresh 0.3% gel; 3—PM in fresh 0.4% gel; and 4—PM in fresh 0.6% gel.

The dependence *I*_φ_ = *f*(*sin*^2^φ) is shown in Figure 3 for gels containing purple membranes (PM). Aqueous suspension of the same PM fraction (line 1) is used as a control. The found *I*_φ_ (0°) = 0 (no light transmitted at fully crossed polarizers: A⊥P, φ = 0) is a criterium that verifies that PM has no measurable optical anisotropy or optical activity, which could contribute to the spontaneous anisotropy Δ*n*_0_ of the composite agarose gels. A freshly prepared 0.3% gel also satisfies this criterium (line 2): *I*_φ_(0°) = 0, which is enough to consider it as being optically isotropic (Δ*n*_0_ = 0). However, *I*_φ_(0°) > 0 is observed (light transmitted through the analyzer even at A⊥P, φ = 0) for composite gels of higher agarose concentrations, which is an indication of optical anisotropy and/or optical activity.

The observed *I*_φ_(0°) > 0 of composite agarose gels can be caused by: (a) Circular birefringence, which appears as a rotation of the polarization plane; (b) linear birefringence of an optically anisotropic medium (Δ*n*_0_ ≠ 0); and/or (c) circular dichroism. The last two effects lead to elliptic polarization of the transmitting light. In order to separate these three effects, it must be taken into account that they affect the slope of the decrossing angle dependence *I*_φ_ = *f*(sin^2^φ) differently. At the elliptic polarization, the slope decreases without breaking the linear dependence, while the optical rotation shifts and deforms the curve with small values of sin^2^φ, but without substantially changing its slope at large sin^2^φ. Figure 3 (lines 3 and 4) shows that the dependence *I*_φ_ = *f*(sin^2^φ) is linear, but has a smaller slope, and the minimal *I*_φ_ value is observed at φ = 0°; this means that there is no optical rotation in gels where *I*_φ_(0°) > 0. Thus, the results show the absence of measurable optical activity (circular birefringence) (see Section 2.7); the light transmitting through the composite gel becomes elliptically polarized. This is an indication of linear birefringence or circular dichroism. Both satisfy the criterion *I*_φ_ (0°) > 0. 

To distinguish the last two effects, it must be considered that the circular dichroism only arises at light absorption. To avoid the possible contribution of PM (incorporated in the gel), the experiments were carried out at a wavelength of λ_0_ = 650 nm, where bR do not have any light absorbance (the circular dichroism of the proteins emerges in UV region). The absorption spectrum of the agarose gels (with or without PM) shows that the light absorbance is negligible at 650 nm (even in the ultraviolet region the large optical density is due to light scattering, while the intrinsic absorbance is negligible); this allows us to reject circular dichroism as a possible source of elliptic light polarization leading to the observed *I*_φ_ > 0 at sin^2^φ = 0 (lines 3 and 4 in Figure 3).

Thus, we can conclude that the light transmission through crossed polarizers (*I*_φ_ > 0 at φ = 0) (and consequently the decrease in the slope of *I*_φ_ *= f*(sin^2^φ) dependence) is caused only by a spontaneous optical anisotropy of the composite gels: a difference Δ*n*_0_ = *n*_0_^//^ − *n*_0_^⊥^ of the refractive indices for the two orthogonal components of the transmitting light, which leads to its elliptical polarization (Section 2.7).

### 2.4. Spontaneous Optical Anisotropy

Once it has been clarified that the cause of the light transmission at the crossed polarizers (*I*_φ_(0°) > 0, Figure 3) is the elliptical polarization caused by linear birefringence, the values of the spontaneous optical anisotropy Δ*n*_0_ can be calculated using Equation (10) from the measured crossed angle dependences *KI*_0_ = *f*(sin^2^φ) by the procedure described in Section 2.2. The results given in Table 1 for 0.3–0.6% agarose gels that were 2 and 24 h old disclosed that the concentration dependence is nonlinear (Section 2.8).

### 2.5. Electrically Induced Optical Anisotropy

The sign of the spontaneous optical anisotropy Δ*n*_0_ can be determined if it is summed up with an anisotropy of a known sign. This information can be obtained by considering the sign of the electrooptical effects of PM in aqueous suspension and in the gel. It is well known that the sign of the electric birefringence Δ*n*_E_ is determined by the orientation (across or along the electric field) of the particles, their geometric form, and the anisotropy of the refractive index of their substance. In the case of PM, the fact that the ratio between the membrane diameter and thickness is more than 100 (800 nm vs. 5 nm) should be considered, while the difference between refractive indices, which are perpendicular and parallel to the membrane plane, hardly exceed 0.1. Therefore, the PM form is a dominant factor that determines the sign of Δ*n*_E_ in the orientation. As is known, the PM have permanent dipole momentum and interfacial electric polarizability, which orient them across or along the field (perpendicularly or parallel to their surfaces in the force lines of the field), respectively, in direct field or in the sinusoidal electric field at kilohertz frequencies [26] (Section 2.16). That means that the sign of EOE Δ*n*_E_ in PM aqueous suspension is positive at a 1 kHz frequency of the applied sinusoidal electric field.

**Figure 4:**
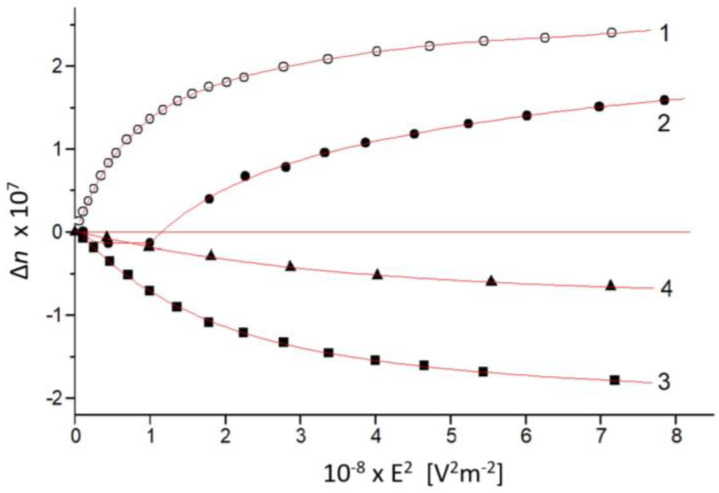
Dependence of the steady-state optical anisotropy Δ*n* on the squared strength *E* of the sinusoidal electric field with a frequency of 1 kHz at a wavelength λ_0_ = 650 nm: 1—PM-water suspension; 2—PM in 0.3% gel, 5 h old; 3—PM in 0.4% gel, 3 h old; and 4—PM in 0.6% gel, 3 h old. The PM concentration in all samples is equal.

Figure 4 shows that the sign of EOE for the anisotropic composite gels is the opposite to that of PM in water suspension. Therefore, the electrically induced anisotropy Δ*n*_E_ has the opposite sign to the spontaneous optical anisotropy Δ*n*_0_ of agarose gel and the absolute value of Δ*n*_E_ is lower compared to Δ*n*_0_. Considering the positive sine (+Δ*n*_E_) at a kilohertz orientation of PM, it can be inferred that the spontaneous optical anisotropy of agarose gel has a negative sign: −Δ*n*_0_ (Section 2.11) This means that the fibers of the gel net have predominant orientation in parallel to the electrode surface.

### 2.6. Kinetics of the Optical Anisotropy

**Figure 5:**
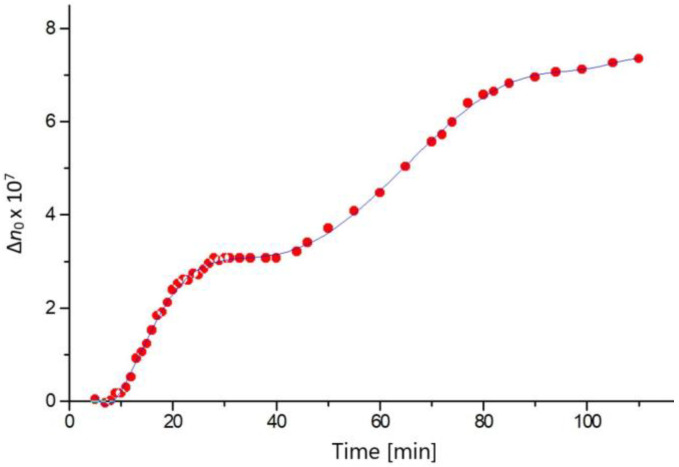
Time dependence of the growth of the spontaneous optical anisotropy Δ*n*_0_ at λ_0_ = 650 nm in 0.6% pure agarose gel.

The data in Table 1 show that the spontaneous optical anisotropy Δ*n*_0_ of the composite agarose gels (containing PM with low constant concentration) is significantly higher at 24 h in comparison with 2 h after gelation. This suggests that the Δ*n*_0_ increase is caused by maturation of the agarose net and can be verified by investigating the dependence of Δ*n*_0_ of pure agarose gel on the time *t* [min]; using pure gel excludes the possible role of PM as an initiator of the maturation, in contrast to the composite gels. The kinetics Δ*n*_0_(*t*) (Figure 5) shows that Δ*n*_0_ of a pure agarose gel has three stages: The fresh gel is optically isotropic (Δ*n*_0_ ≈ 0) at nearly 10 min after sol-gel transition (lag-phase); then, the anisotropy emerges and its value increases with time in two stages (Section 2.10).

### 2.7. Optical Activity of Agarose Gels

The conclusion that the agarose composite gels have no optical activity (Section 2.3) seemingly contradicts to the literature data that the agarose is optically active and the activity alters at the sol–gel transition [30]. Our inference is based on the fact that the intensity of the transmitted light at crossed polarizers (A⊥P) is practically zero (*I*_φ_ = 0 at φ = 0); this means that there is no measurable optical rotation that can be caused by circular birefringence; the contribution of the circular dichroism is excluded due to the absence of intrinsic light absorption at the wavelength λ_0_ = 650 nm used. Such an effect (*I*_φ_ > 0 at φ = 0) can only be registered if the intensity of the light transmitted through the analyzer A exceeds a certain limit defined by the apparatus sensitivity; this limit corresponds to the rotation of the polarization plane with φ ≥ 0.1° in our case. However, according to the literature, the rotation is considerably lower for agarose gels of the same concentration. For example, the optical rotation increases from −0.018° to −0.027° at the sol–gel transition in the 0.3% water solution of agarose [31]. This explains the fact that no optical rotation was observed in our experiments. Moreover, the optical activity of PM should not alter at the sol–gel transition of agarose because there are no any structural changes in bacteriorhodopsin in the 20–50 °C temperature range at the cooling after addition of PM suspension to agarose solution [32].

### 2.8. Nonlinear Concentration Dependence

Table 1 shows that the spontaneous anisotropy Δ*n*_0_ in composite gels (at constant concentration of the incorporated PM) increases with agarose concentration, which correlates with the data for clear agarose gels in Reference [22]. This result may seem trivial, since the average refraction index *n*_0_ = (*n*_0_^//^ + *n*_0_^⊥^)/2 of the gel increases with agarose concentration *c*. However, the Δ*n*_0_ increase is not proportional to *c* and is even equal to zero in freshly prepared 0.3% gel. In principle, such behavior can be related to the dependence of the increment of the refractive indices *dn/dc* = (*n*_g_ − *n*_w_)/*c*, where *n*_g_ and *n*_w_ are the refractive indices of gel and water, respectively. However, at such low agarose concentrations *c* = 0.3–0.6%, the dependence *n*_g_ − *n*_w_ = *f*(*c*) is linear for other polymers [33] and it is unlikely to presume an exception for agarose gels.

Another, more likely explanation of the non-linear concentration dependence Δ*n*_0_ = *f*(*c*) lies in the different packing of agarose fibers at varying agarose concentrations. It can be expected that, at higher concentrations, the length of conjugated regions of the fibers grows, as well as the number of agarose molecules in one conjugate. Since such conjugates are greatly anisodiametric [34], the optical anisotropy Δ*n*_0_ grows at their orientation. Thus, the second explanation lies in the dependence of anisodiametricity and density of agarose molecule conjugates on the total agarose concentration *c*, which results in the non-linearity of the concentration dependence Δ*n*_0_ = *f*(*c*). This conclusion is supported by electron microscopy studies showing that, with an agarose concentration increase, the fibrous regions become more densely packed [35,36,37].

### 2.9. Contribution of PM to the Spontaneous Anisotropy 

It can be assumed that the spontaneous optical anisotropy Δ*n*_0_ of the composite gel is related to the presence of the plate-like PM: if they are immobilized, they would become partly oriented during the deformation of the gel net, and this will result in the appearance of Δ*n*_0_. However, the optical anisotropy emerges even when there are no PMs in the gel (Figure 5). The comparison of composite gels with pure gels shows that, in a certain period, the Δ*n*_0_ values of the two types of gels become almost equal; this proves that the contribution of PM to the spontaneous optical anisotropy observed in composite gels is negligible. That fact could be explained as follows: The PM concentration in composite gels was chosen so that the electrically induced optical anisotropy Δ*n*_E_, even at a high degree of orientation, is commensurable with the spontaneous anisotropy Δ*n*_0_ of the pure gel; this is clear from the comparison of Δ*n*_E_ at a high field strength (Figure 4, curve 1), which is less than Δ*n*_0_ of fresh 0.4% gel (Table 1). Our electrooptical measurements show that the greater part of PM retains its orientational mobility in gels with an agarose concentration of 0.3–0.4% and the immobilized membranes are predominantly chaotically oriented. Therefore, the PM contribution to the spontaneous anisotropy Δ*n*_0_ is about two orders of magnitude lower than electrically induced anisotropy Δ*n*_E_ at full orientation in aqueous suspension of PM with the same concentration. Thus, the intrinsic gel anisotropy is absolutely dominant in comparison to the relatively small anisotropy caused by the partial orientation of immobilized PM.

### 2.10. Gel Deformation and Fiber Orientation

The spontaneous optical anisotropy Δ*n*_0_ is a manifestation of the anisotropic orientation of agarose fibers, which can occur upon deformation of the gel net caused by inhomogeneous and anisotropic specimen cooling. Owing to the appearance of intermolecular associates (before the gel net formation), the viscosity of the agarose solution increases strongly and this hinders its convection in the electrooptical cell; the cooling is then mainly due to heat conduction, which is much slower then the convective heat transfer. Due to the high heat conductivity of the metal electrodes, the electrode zone is quickly cooled down and gelation emerges there first, while the central zone remains as an agarose solution. It might suggest that the agarose fibers are absorbed even more on the electrodes (due to the effect of electron mirror which increases the attraction) than on the dielectric surface, as in the case of the adsorption of cellulose on alumina particles [38], and this induces a predominant orientation of the fibers in parallel to the electrode surface at the deformation of the gel net, caused by thermal constriction of the gel; the orientation spreads in the gel interior as the temperature there decreases. It is obvious that the agarose concentration should be high enough to achieve the distant orientational order macroscopically manifested. Indeed, Δ*n*_0_ arises faster in gels with higher agarose concentrations. At a low concentration, the predominant orientation is only achieved after the formation of a sufficient number of intermolecular contacts; this process requires an additional lapse of time (Table 1).

To verify the above suggestion, we can make the following assessment. When the temperature is lowered from 50 °C to 20 °C, the gel volume of the agarose solution/gel decreases with 1%, while its level in our electrooptical cell decreases with 0.3 mm. The refractive index increment is *dn/dc* = 0.14 at 633 nm [39]; this value means that the refractive index *n* of 0.6% gel is only higher with 8 × 10^−4^ than that of water at 650 nm. Assuming that the partial orientation of the agarose fibers (at cell level lowering with 1%) is equivalent to the full orientation of 1/3 of 1% of the fibers, the refractive index *n*^⊥^ (perpendicular to the electric field direction, in parallel to the electrode surface) would increase with 2.7 × 10^−6^, and the perpendicular *n*_0_^//^ would decrease by the same value. This shows that, at a thermal constriction, the maximal value, which is Δ*n* = *n*^//^ − *n*^⊥^, might reach −5.4 × 10^−6^, is an order higher than the experimentally found value of Δ*n*_0_. That is, the anisotropic deformation of the gel net caused by inhomogeneous cooling is the real cause of the partial orientation of the agarose fibers, which is manifested by the spontaneous optical anisotropy of the gel specimen. We suggest that this process corresponds to the first step of the kinetic curve (Figure 5). The second step is probably related to the evaporation of part of the free water in the gel, which causes a further volume decrease and deformation of the gel net. The slow changes in the net structure also likely provides a contribution over time [40]; the additional formation of intermolecular bonds in the agarose gels can be described by the term “maturing” and by employing an analogy with gelatine gel [41].

### 2.11. Electrooptical Effects in Composite Gels

Depending on the sign and absolute values |*n*| of the spontaneous Δ*n*_0_ and electrically induced *n*_E_ optical anisotropy Δ*n* = Δ*n*_0_ + Δ*n*_E_ in the composite agarose gels, EOE Δ*I*_E_ might have either a positive or negative sign. At a negative Δ*n*_0_, the positive Δ*n*_E_ results in a decrease in the total phase retardation δ = −δ_0_ + δ_E_ and thus to the reduction of transmitted light intensity *I*_E_ passing through the analyzer, i.e., it manifests as negative EOE Δ*I*_E_ when +Δ*n*_E_ < |−Δ*n*_0_|; then Δ*n* is negative (Figure 4, curves 3 and 4). EOE is deformed when the positive Δ*n*_E_ is bigger than the absolute value of the negative Δ*n*_0_; then the moment value Δ*I*_t_ of the transient EOE Δ*I*_t_(*t*) at first decreases with time *t* to zero but then increases because of an increase in the degree of orientation of the incorporated PM. In the last case (+Δ*n*_E_ > |−Δ*n*_0_|) the dependence of the steady-state EOE Δ*I*_s_ on the field strength *E* is similar: At low degrees of orientation (γ*E*^2^ < *kT*), Δ*I*_s_ is negative but becomes positive at increasing of *E*. We observed this in gels with low agarose concentrations (Figure 4, curve 2), where the intrinsic gel anisotropy is negative, but has a lower absolute value than the electrically induced one, i.e., when Δ*n*_E_ > |−*n*_0_| the field strength dependence Δ*n*_s_ = *f*(*E*^2^) of the steady-state optical anisotropy Δ*n*_s_ is deformed being negative at low degrees of PM orientation and positive at high degrees (γ*E*^2^ > *kT*). In the above cases the electrically induced anisotropy Δ*n*_E_ is positive (Figure 4, curve 1) because at kilohertz frequencies PM are oriented in parallel to the electric field. However, Δ*n*_E_ is negative in direct field because of perpendicular orientation of the PM (Section 2.16); then the moment Δ*I*_t_ and steady-state Δ*I*_s_ values of EOE are always positive independently on the ratio Δ*n*_E_/Δ*n*_0_ because Δ*n* = |−Δ*n*_E_| + |−Δ*n*_0_|.

### 2.12. Inhomogeneity of Agarose Gels

The dependence of the spontaneous anisotropy Δ*n*_0_ on the agarose concentration *c* in pure gels investigated by Stellwagen [22] was found to be linear for a certain point in a great number of gels, but the average value of Δ*n*_0_ for each gel manifests nonlinearity at concentrations below 0.6% like Δ*n*_0_ = *f*(*c*) of our composite gels. This may be due to the choice of points of the Δ*I*_E_ measured, whose values are used for averaging. This a problem that arises at a narrow beam like the laser one. As we used a monochromator, our light beam had a large cross section and it passed through the whole volume of the electrooptical cell; this means that our value of Δ*n*_0_ was averaged over all gel regions. The 5 cm optical path additionally raises five times (in comparison with 1 cm path cell in References [16,17,18,19,20,21,22]) the observed volume of the gel. That is why we believe that the value of Δ*n*_0_ measured in our electrooptical cell is a representative average quantity.

### 2.13. Structural Anisotropy of Agarose Gels

The results discussed above disclose that the spontaneous optical anisotropy Δ*n*_0_ of the composite gels is determined by the deformation of the gel net, and the contribution of the incorporated PM is negligible. This means that the conclusions can be spread on agarose gels, which do not contain colloid particles as the circumstantially investigated in Stellwagens’ works [16,17,18,19,20,21,22]. Other than the refractive index anisotropy, the structural anisotropy of the agarose gels manifest itself in anisotropy of other properties of the agarose gels: Optical [42,43,44], electrical [45,46], mechanical [47], and magnetic [48,49,50,51].

### 2.14. Surface Electrostatic Potential

The bR monomer in Figure 1 and the bR trimers (3bR) in Figure 2 are colored according to the electrostatic potential (red—negative and blue—positive) on a surface delineated by the closest access of a sphere with a size of a water molecule to the surface atoms of the protein globule (the distance is determined by the van-der-Waals radii of the atoms). The surface separates the internal (hydrophobic core) and external (aqueous) mediums of the bR and 3bR, which are assumed to be unstructured continuums with relative dielectric permittivity ε equal to 4 and 80, respectively. The local surface potential is computed as the superposition of the elementary potentials created by the coulomb or partial charges of all atoms of the bR and 3bR, taking into account their distance to a given point on the surface (determined by their 3D coordinates) and the permittivity ε of the inner and outer mediums. The negative and positive coulomb charges are determined by the NH_3_^+^ and COO^−^ groups whose charge is caused by the associated or dissociated H^+^ at pH 6.0 (pH of the aqueous solution in the experiment); these charges are located on the surface of the protein globule, where the most chargeable hydrophilic aminoacid residues are predominantly disposed (the single COOH and NH_2_ groups located inside are uncharged because of the low permittivity of the hydrophobic protein core and the inaccessibility to H_2_O molecules of the external medium). The partial charges in bR and 3bR are determined by polarization of the covalent bonds due to the different electron affinity of their atoms. The electrostatic potential is calculated in *kT/e* units, where *k*—Boltzmann constant [J/K], *T*—absolute temperature [K], and |*e*|—charge of the electron [C]; *kT*/*e* [J/C] = 25.69 mV at 25 °C). The potential on the surface of the bR and 3bR is visualized by coloring according to its value and sign (negative or positive).

### 2.15. Sieving Resolution

The presence of gel optical anisotropy is an indication that the statistically averaged pore form is anisodiametric. We can suppose that the predominant orientation of the gel net fibers in one direction would facilitate the macromolecules migration in the direction of elongated axis pores as they move further without hindering. This probably affects the gel resolution in the case of globular proteins. The different pore anisodiametricity along the depth of the electrophoretic plate results in band broadening. The influence of gel anisotropy on its sieving properties in the case of (semi)flexible chains as dsDNA, ssDNA, RNA, polypeptides and denaturated proteins should depend on the contour length (molecular mass) and flexibility of the chain and the agarose concentration, as well as on the field strength (voltage) and the period of forward–reverse pulses when a field inversion gel electrophoresis technique is applied.

The spontaneous structural anisotropy of agarose gels probably occurs in all cases when they contact a wall of different materials, particularly in the apparatus for horizontal gel electrophoresis, although the optical anisotropy cannot be registered then because the linearly polarized light beam passes vertically, but the predominant fibers orientation is in the horizontal plane, i.e., the gel is isotropic in respect to the plane of light polarization. However, this vertical anisotropy of the gel net leads to a decrease in the resolution because of band broadening caused by the different velocity of macromolecules migration. 

### 2.16. Electrically Regulated Sieving

The use of plate-like colloid particles having transversal permanent dipole moment *p* and electric polarizability γ allows for the modulation of the sieving properties of composite gels by different particle orientation depending on the frequency ν and strength *E* of the applied direct (d.c.) or alternating (a.c.) electric field. The orientational behavior of the purple membranes (PM) is determined by the competition of the torques *pE* ⊥ γ*E*^2^, which are determined by the orthogonally oriented permanent *p* and induced γ*E* dipole moments. The transversal orientation of the permanent dipole is determined by the electric asymmetry of PM (Section 2.1, Figure 2); the value of *p* is proportional to the area of PM. The interface electric polarizability γ is determined by the polarization of the counterions, which surround PM to compensate for their negative net charge (curve 3 in Figure 1); the value of γ is proportional to the surface charge density (the number of charges per unit area) and the distance on which the counterions can migrate for a half-period of the applied sinusoidal electric field. 

**Figure 6:**
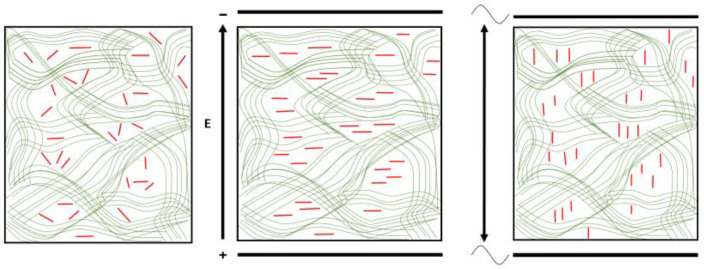
Imaginary picture of the composite agarose gel with incorporated PM, which are shown schematically as rods (diametrical cross section of the membrane plane) oriented chaotically (the left picture), perpendicularly to the direct electric field (the medium picture) and along the field direction in the kilohertz sinusoidal electric field (the right picture).

In the direct electric field with low strength *E* (then *p* > γ*E* and *pE* > γ*E*^2^), PM are oriented transversely (perpendicularly) to the field direction (Figure 6), but in high voltage fields, the orientation becomes along the field under the action of the stronger torque γ*E*^2^ > *pE* (then the induced dipole moment prevails: γ*E* > *p*). On the contrary, in a sinusoidal electric field with a kilohertz frequency, PM are orientated along (in parallel) the field under the action of the torque γ*E*^2^ at all field strengths (the value of *E* determines only the degree of orientation from low to full) because PM are unable to follow the changing field direction (then the permanent dipole remains hidden). At low frequencies ν and low field strength (*p* > γ*E* and *pE* > γ*E*^2^) of the applied sinusoidal electric field, PM can still follow the alternating field direction and then they are oriented predominantly transversely (as in the case of direct field), but as the frequency ν increases, the orientation becomes along the field; the critical frequency (in the region of 100 Hz) depends on the ratio of the rotational diffusion coefficient *D*_r_ (determined by PM size and viscosity of the medium) and the half-period *T*_E_/2 [s] of the sinusoidal field (*T*_E_/2 = 0.5 ms at ν = 1 kHz).

Thus, in direct fields with low strength (usually used for continuous or pulse gel electrophoresis), the transverse orientation of PM plates reduces the effective pore size and by that hinder the migration of the larger macromolecules, but do not disturb the smaller ones; this leads to an increase in the sieving resolution. In the combined electric field with direct and kilohertz components (the first moves the macromolecules, the second orients the incorporated PM), the migration velocity of the macromolecules will depend on the ratio of the two field components, in this case the saving depends on the degree of PM orientation. Moreover, PM has an additional peculiarity. It has the form of the cylindrically bent elliptical disk [52]; this means that, in the direct field, all the membranes are oriented uniformly with their curvature to the field and this hinders or facilitates the macromolecule motion depending on the direction of their migration.

## 3. Conclusions

The investigation of the composite agarose gels with incorporated bacteriorhodopsin (bR) purple membranes (PM), carried out by the method of electric birefringence, disclosed that the gels manifest complex optical anisotropy with two components: Spontaneous and electrically induced. The procedure developed on the base of the classical birefringence theory allows for a quantitative separation of the two components.

The spontaneous anisotropy emerges in both pure and composite agarose gels; in the last case the incorporated PM do not contribute noticeably to its value. This type of intrinsic anisotropy emerges because of predominant orientation of the agarose fibers at deformation of the gel net caused by the thermal constriction at the cooling. The spontaneous anisotropy increases with the agarose concentration and the time; at low concentration its kinetics shows three stages: lag phase ant two step increments.

The induced anisotropy of the composite agarose gel is caused by orientation of the incorporated PM at applying of electric field. The orientation is due to the negative net charge and electric asymmetry of the lipid-protein complexes of PM. The computation by protein electrostatics discloses that at pH 6 the bR macromolecules are positively charged but the negative net charge of PM is due to the lipid molecules associated to bR trimers. The electrostatic potential on the two surfaces of the PM is asymmetrical (more negative on the cytoplasmic surface) because of the specific 3D location of the charged aminoacid residues of the bR protein globules.

The surface electric charge and its asymmetry determine an induced and permanent dipole moments, which orient the PM along or across the lines of force at applying of sinusoidal electric field with kilohertz frequency or direct electric field, respectively. This peculiarity of PM opens a perspective to modulate sieving properties of composite agarose gels by electric modulation of the effective pore size of the gel net by control of the direction and degree of orientation of the incorporated PM.

## 4. Materials and Methods

### 4.1. Materials

PM were kindly granted to us by Prof. E. Neumann. The suspension was fractionated (after ultrasonic disaggregation for 3 sec at 22 kHz) with a centrifuge Janetzki T-24 (Germany) at 19,500 rpm for 15 min and 10,000 rpm for 5 min for the separation of small and large membranes. The relaxation time of the disorientation after the orientation in electric fields with various intensity showed that the obtained suspension is almost monodispersed. The size of PM, calculated from the relaxation time, after being filed as switching out, is equal to a disk with a diameter of *B* = 0.8 μm.

The gel was prepared by heating water LE agarose suspension for 10 min in a boiling water bath and by the subsequent cooling down to 50 °C and addition of the PM suspension, which had been preliminarily heated to the same temperature. The final agarose concentration varied from 0.3% to 0.6%, and the PM concentration in the gel was 1.5 μM (40 μg/mL) bacteriorhodopsin. The average distance between the mass centers of two neighboring membranes, estimated from the concentration and the size of PM, was 4.0 μm; this ensured that the orientation of PM plates were independent of the neighbors. The electrooptical measurements began approximately 30 min after gel cooling.

### 4.2. Experimental Set-Up 

**Figure 7:**
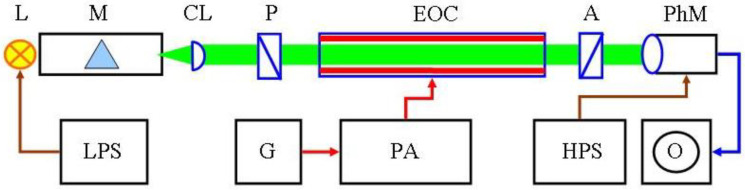
Schematic diagram of the set-up used for electric birefringence: L—halogenous lamp 12 V, 100 W; LPS—low-voltage power supply unit; M—prism monochromator; CL—cylindrical lens; P—polarizer; EOC—electrooptical cell; A—analyzer; PhM—photomultiplier; HPS—high-voltage power supply unit; O—digital oscilloscope; G—functional generator; PA—wide band power amplifier. The coloured components are: lamp (yellow); light beam (green), metal electrodes (two thick red lines in EOC); electric impulses (red lines); electrooptical signal (blue line), low- and high-voltage direct current (brown lines).

The birefringence set-up used (Figure 7) is built on the base of the prism spectrophotometer LOMO (Russia) and dichroic polarizer P and analyzer A; the plane of the polarization is inclined at 45° to the electric field. The apparatus has a crossing coefficient *I*^⊥^/*I*^//^ = 1 × 10^−4^ and sensitivity Δ*n*_min_ = 1 × 10^−8^ at the used λ_0_ = 650 nm. The electrooptical cell consists of a quartz cuvette and two parallel vertical electrodes with dimensions 50 × 47 × 2 mm fastened in a teflon holder. The optical path of the cell was 50 mm and the interelectrode distance was 3.0 mm. The gel sample had the dimensions: 30 mm height, 50 mm length and 3.0 mm thickness. Sinusoidal a.c. voltage up to 140 V, with a frequency of 1 kHz, generated by the functional generator Wavetek-125 and amplified by a wide band amplifier Optimation PA-25 (USA), was applied to the electrodes. The EOE Δ*I*_E_ [mV] were registered by digital oscilloscope Fillips at appropriate electric impulses with a strength *E* and millisecond length, which allow for reaching the steady-state orientation of the PM in the agarose gel.

### 4.3. Protein Electrostatics

The surface electrostatic potential of purple membrane lamella (PM) and bacteriorhodopsin globules in monomeric (bR) and trimeric (3bR) form was computed with the programs for protein electrostatics PHEMTO [53], Propka [54,55] and Bluues [56] using the atomic coordinates of the native structure of the bR macromolecule (Protein data bank ID: 1BRR), and then visualized by Chimera [57]. The coordinates of the lipid molecule adjacent to the protein trimer 3bR were obtained with the program for molecular dynamics Charmm GUI membrane builder [58].

## Figures and Tables

**Table 1:** Spontaneous optical anisotropy of composite agarose gels at λ_0_ = 650 nm with different agarose concentrations at a constant concentration of purple membranes.

Concentration	0.3%		0.4%		0.6%	
**Age**	2 h	24 h	2 h	24 h	2 h	24 h
**Δ*n* × 10^7^**	0.1	2.1	4.6	5.1	7.5	8.2

## Data Availability

Not applicable.
